# Supplementary material for: Ret receptor tyrosine kinase sustains proliferation and tissue maturation in intestinal epithelia
Source: EMBO J. 2017 Sep 12;36(20):3029–45. doi: 10.15252/embj.201696247 (PMC5641678; doi:10.15252/embj.201696247)
Supplement: Supplementary file 1 — Appendix [file EMBJ-36-3029-s001.pdf]

Full Genotypes

Figure 1

(B, D) w/w; Ret-Gal4/UAS- UAS-Flybow 1.1; +/+

(C) w/w; Ret-Gal4/UAS-StGFP; +/+

(E) w, Su(H)^{GBE}-LacZ/w; esg-Gal4^{NP7397}, UAS-GFP, tub-Gal80^{TS}/+; +/+

(F, G) w, Su(H)^{GBE}-LacZ/w; Ret-Gal4/UAS-StGFP; +/+

Figure 2

(A, C, F) Control: w, Su(H)^{GBE}-LacZ/w; esg-Gal4^{NP7397}, UAS-GFP, tub-Gal80^{TS}/+; UAS-Dicer2/+
Ret-RNAi: w, Su(H)^{GBE}-LacZ/w; esg-Gal4^{NP7397}, UAS-GFP, tub-Gal80^{TS}/+; UAS-Ret$^{RNAi\ GD843}$, UAS-Dicer2/+

(B) Control: w, hs-flp,tub-Gal4, UAS-GFP/w, Su(H)^{GBE}-LacZ; tub-Gal80, FRT40A/FRT40A; +/+
Ret-RNAi MARCM: w, hs-flp,tub-Gal4, UAS-GFP/w, Su(H)^{GBE}-LacZ; tub-Gal80, FRT40A/FRT40A; UAS-Ret$^{RNAi\ GD843}$, UAS-Dicer2/+
Ret^{KO} MARCM: w, hs-flp,tub-Gal4, UAS-GFP/w, Su(H)^{GBE}-LacZ; tub-Gal80, FRT40A, Ret^{KO}/FRT40A; +/+

(D, G) Control: w/w; +/+; +/+
Heterozygotes: w/w; Ret^{KO}/+; +/+
Mutants: w/w; Ret^{KO}/Ret^{KO}; +/+

(E) Control: w, Su(H)^{GBE}-LacZ/w; esg-Gal4^{NP7397}, UAS-GFP, tub-Gal80^{TS}/+; +/+
UAS-Ret: w, Su(H)^{GBE}-LacZ/w; esg-Gal4^{NP7397}, UAS-GFP, tub-Gal80^{TS}/+; UAS-Ret-3xFLAG-6xHis/+

(H) Control: w, hs-flp,tub-Gal4, UAS-GFP/w, Su(H)^{GBE}-LacZ; tub-Gal80, FRT40A/FRT40A; +/+
UAS-Ret: w, hs-flp,tub-Gal4, UAS-GFP/w, Su(H)^{GBE}-LacZ; tub-Gal80, FRT40A/FRT40A; UAS-Ret-3xFlag-6xHis/+

Figure 3

(A) Control: w, Su(H)^{GBE}-LacZ/w; esg-Gal4^{NP7397}, UAS-GFP, tub-Gal80^{TS}/+; +/+
Mitogens:
(upd1) w, Su(H)^{GBE}-LacZ/w; esg-Gal4^{NP7397}, UAS-GFP, tub-Gal80^{TS}/UAS-upd1; +/+
w, Su(H)^{GBE}-LacZ/w; esg-Gal4^{NP7397}, UAS-GFP, tub-Gal80^{TS}/UAS-upd1; UAS-Ret$^{RNAi\ GD843}$, UAS-Dicer2/+
(sspitz) w, Su(H)^{GBE}-LacZ/w; esg-Gal4^{NP7397}, UAS-GFP, tub-Gal80^{TS}/+; UAS-sspitz/+
w, Su(H)^{GBE}-LacZ/w; esg-Gal4^{NP7397}, UAS-GFP, tub-Gal80^{TS}/+; UAS-sspitz/UAS-Ret$^{RNAi\ GD843}$, UAS-Dicer2
(wg) w, Su(H)^{GBE}-LacZ/w; esg-Gal4^{NP7397}, UAS-GFP, tub-Gal80^{TS}/UAS-wg::HA; +/+
w, Su(H)^{GBE}-LacZ/w; esg-Gal4^{NP7397}, UAS-GFP, tub-Gal80^{TS}/UAS-wg::HA; UAS-Ret$^{RNAi\ GD843}$, UAS-Dicer2/+
(sgg^{DN}) w, Su(H)^{GBE}-LacZ/w; esg-Gal4^{NP7397}, UAS-GFP, tub-Gal80^{TS}/+; UAS-sgg.A81T/+
w, Su(H)^{GBE}-LacZ/w; esg-Gal4^{NP7397}, UAS-GFP, tub-Gal80^{TS}/+; UAS-sgg.A81T/ UAS-Ret$^{RNAi\ GD843}$, UAS-Dicer2

(B) Control: w, Su(H)^{GBE}-LacZ/w; esg-Gal4^{NP7397}, UAS-GFP, tub-Gal80^{TS}/+; +/+
UAS-Ret: w, Su(H)^{GBE}-LacZ/w; esg-Gal4^{NP7397}, UAS-GFP, tub-Gal80^{TS}/+; UAS-Ret-3xFLAG-6xHis/+

(C) Control: w, Su(H)^{GBE}-LacZ/w; esg-Gal4^{NP7397}, UAS-GFP, tub-Gal80^{TS}/+; +/+

UAS-Ret: w, Su(H)^{GBE}-LacZ/w; esg-Gal4^{NP7397}, UAS-GFP, tub-Gal80^{TS}/+; UAS-Ret-3xFLAG-6xHis/+

+ dsh^{DN}: w, Su(H)^{GBE}-LacZ/w; esg-Gal4^{NP7397}, UAS-GFP, tub-Gal80^{TS}/+; UAS-dshΔB212/UAS-Ret-3xFLAG-6xHis

+pan^{DN}: w, Su(H)^{GBE}-LacZ/w; esg-Gal4^{NP7397}, UAS-GFP, tub-Gal80TS/+; panDN/ UAS-Ret-3xFLAG-6xHis

(D) *Control: w, Su(H)^{GBE}-LacZ/w; esg-Gal4^{NP7397}, UAS-GFP, tub-Gal80^{TS}/+; +/+*

UAS-wg: w, Su(H)^{GBE}-LacZ/w; esg-Gal4^{NP7397}, UAS-GFP, tub-Gal80^{TS}/+; UAS-wg-HA/+

Ret-RNAi: *w, Su(H)^{GBE}-LacZ/w; esg-Gal4^{NP7397}, UAS-GFP, tub-Gal80^{TS}/+; UAS-Ret$^{RNAi\ GD843}$, UAS-Dicer2/+*

UAS-Ret: w, Su(H)^{GBE}-LacZ/w; esg-Gal4^{NP7397}, UAS-GFP, tub-Gal80^{TS}/+; UAS-Ret-3xFLAG-6xHis/+

(E) *Control: w; wg-Gal4^{KO}/tub-Gal80^{TS}; UAS-mCD8-GFP /+*

UAS-Ret: w; wg-Gal4^{KO}/tub-Gal80^{TS}; UAS-mCD8-GFP /UAS-Ret-3xFLAG-6xHis/+

Figure 4

(A) *UAS-Ret: w, Su(H)^{GBE}-LacZ/w; esg-Gal4^{NP7397}, UAS-GFP, tub-Gal80^{TS}/+; UAS-Ret-3xFLAG-6xHis/+*

UAS-Ret: w, Su(H)^{GBE}-LacZ/w; esg-Gal4^{NP7397}, UAS-GFP, tub-Gal80^{TS}/+; UAS-Ret^{K805M} -V5/+

(B) *Control: w, Su(H)^{GBE}-LacZ/w; esg-Gal4^{NP7397}, UAS-GFP, tub-Gal80^{TS}/+; +/+*

UAS-Ret: w, Su(H)^{GBE}-LacZ/w; esg-Gal4^{NP7397}, UAS-GFP, tub-Gal80^{TS}/+; UAS-Ret-3xFLAG-6xHis/+

(C) *Control: w, Su(H)^{GBE}-LacZ/w; esg-Gal4^{NP7397}, UAS-GFP, tub-Gal80^{TS}/+; +/+*

UAS-Ret: w, Su(H)^{GBE}-LacZ/w; esg-Gal4^{NP7397}, UAS-GFP, tub-Gal80^{TS}/+; UAS-Ret-3xFLAG-6xHis/+

+ Src42A^{RNAi}: w, Su(H)^{GBE}-LacZ/w; esg-Gal4^{NP7397}, UAS-GFP, tub-Gal80^{TS}/UAS-Src42A$^{RNAi\ kk100708}$; UAS-Ret-3xFLAG-6xHis/+

+ Src64B^{RNAi}: w, Su(H)^{GBE}-LacZ/UAS-Src64B$^{RNAi\ GD35252}$; esg-Gal4^{NP7397}, UAS-GFP, tub-Gal80^{TS}/+; UAS-Ret-3xFLAG-6xHis/+

(D, E) *Control: w, Su(H)^{GBE}-LacZ/w; esg-Gal4^{NP7397}, UAS-GFP, tub-Gal80^{TS}/+; +/+*

Src42A^{CA}: w, Su(H)^{GBE}-LacZ/w; esg-Gal4^{NP7397}, UAS-GFP, tub-Gal80^{TS}/+; UAS-Src42A^{CA}/+

+ Ret^{RNAi}: w, Su(H)^{GBE}-LacZ/w; esg-Gal4^{NP7397}, UAS-GFP, tub-Gal80^{TS}/+; UAS-Src42A^{CA}/UAS-Ret$^{RNAi\ GD843}$, UAS-Dicer2

(F) *Control: w, Su(H)^{GBE}-LacZ/w; esg-Gal4^{NP7397}, UAS-GFP, tub-Gal80^{TS}/+; +/+*

UAS-Ret: w, Su(H)^{GBE}-LacZ/w; esg-Gal4^{NP7397}, UAS-GFP, tub-Gal80^{TS}/+; UAS-Ret-3xFLAG-6xHis/+

(G, H) *UAS-Ret: w, Su(H)^{GBE}-LacZ/w; esg-Gal4^{NP7397}, UAS-GFP, tub-Gal80^{TS}/+; UAS-Ret-3xFLAG-6xHis/+*

UAS-Ret: w, Su(H)^{GBE}-LacZ/w; esg-Gal4^{NP7397}, UAS-GFP, tub-Gal80^{TS}/UAS-dFAK; UAS-Ret-3xFLAG-6xHis/+

Figure EV1

(A) *Control: w, hs-flp,tub-Gal4, UAS-GFP/w, Su(H)^{GBE}-LacZ; tub-Gal80, FRT40A/FRT40A; +/+*

Ret^{RNAi}: w, hs-flp,tub-Gal4, UAS-GFP/w, Su(H)^{GBE}-LacZ; tub-Gal80, FRT40A/FRT40A; UAS-Ret$^{RNAi\ GD843}$, UAS-Dicer2/+

	<u>Ret^{KO}</u>: w, hs-flp,tub-Gal4, UAS-GFP/w, Su(H)^{GBE}-LacZ; tub-Gal80, FRT40A, Ret^{KO}/FRT40A; +/+
(B)	*<u>Control</u>: w, Su(H)^{GBE}-LacZ/w; esg-Gal4^{NP7397}, UAS-GFP, tub-Gal80^{TS}/+; UAS-Dicer2/+*
	<u>Ret^{RNAi}</u>: w, Su(H)^{GBE}-LacZ/w; esg-Gal4^{NP7397}, UAS-GFP, tub-Gal80^{TS}/+; UAS-Ret$^{RNAi\ GD843}$, UAS-Dicer2/+
(C, D)	*<u>Control</u>: w/w; +/+; +/+*
	<u>Ret^{KO}</u>: w/w; Ret^{KO}/Ret^{KO}; +/+
