# Supplementary material for: Ret receptor tyrosine kinase sustains proliferation and tissue maturation in intestinal epithelia
Source: EMBO J. 2017 Sep 12;36(20):3029–45. doi: 10.15252/embj.201696247 (PMC5641678; doi:10.15252/embj.201696247)
Supplement: Supplementary file 2 — Expanded View Figures PDF [file EMBJ-36-3029-s002.pdf]

Expanded View Figures

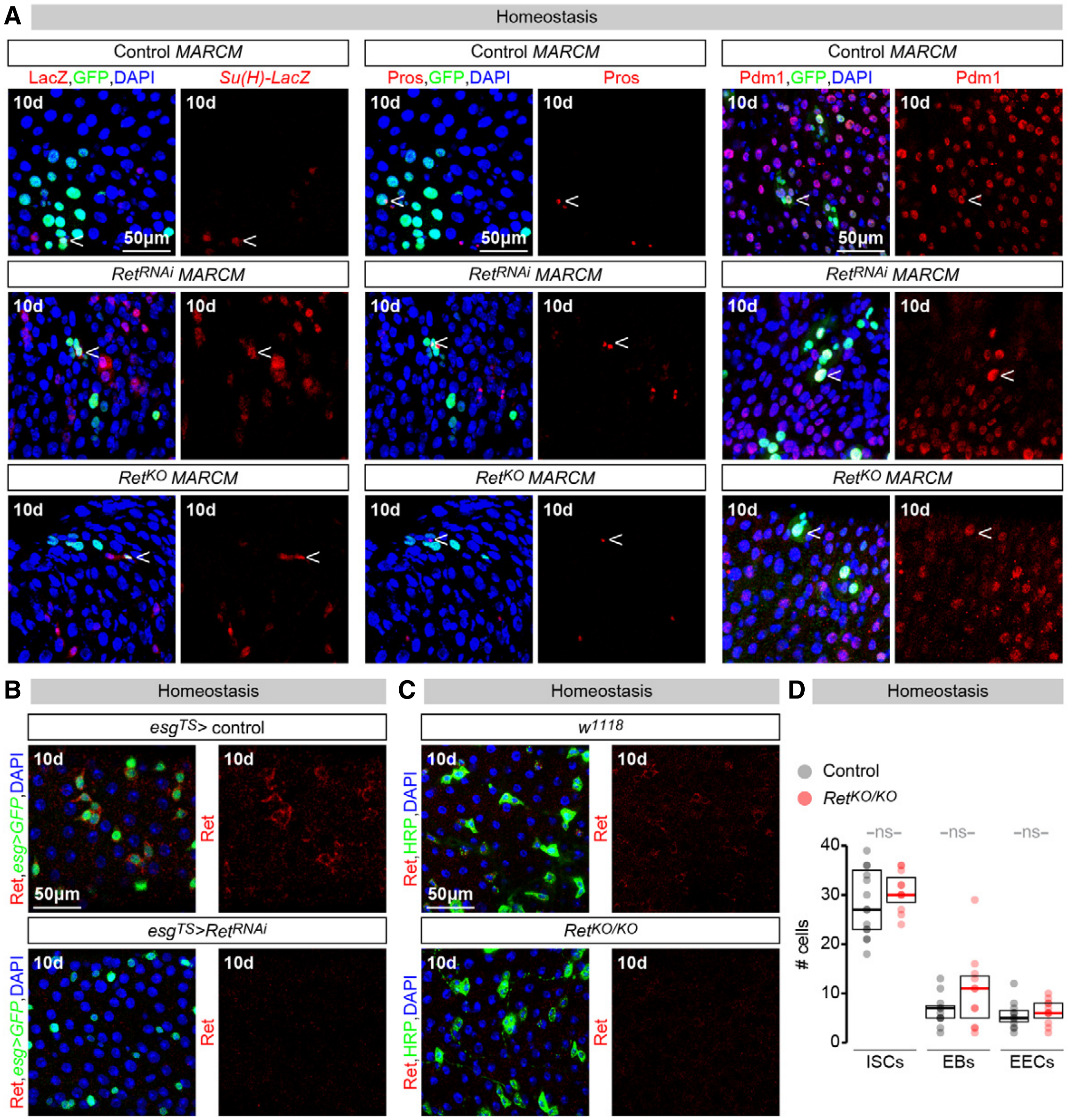

Figure EV1. Cell fate and Ret expression analyses in *Ret* mutants/knockdowns.

A Like cells of control clones, cells of MARCM clones expressing a *Ret-RNAi* transgene (*Ret*^{RNAi} *MARCM*) or entirely lacking *Ret* expression (*Ret*^{KO} *MARCM*) are positive for a *Su(H)-LacZ* (an EB marker), Pros (an EE marker) and Pdm1 (an EC marker), indicating that loss of *Ret* does not compromise the ability of intestinal progenitors to differentiate.

B Ret immunostainings of adult midguts indicate that adult-specific downregulation of *Ret* in intestinal progenitors (achieved by *esg-Gal4, tub-Gal80TS* expression of a *Ret-RNAi* transgene) effectively downregulates Ret protein in these cells (labelled with *esg-Gal4*-driven GFP).

C Ret immunostaining is also absent from the adult intestinal progenitors of *Ret*^{KO} mutants [labelled with anti-horseradish peroxidase (HRP)].

D Quantifications of different midgut epithelial cell types (based on the markers used in previous panels) in 4-day-old control and *Ret*^{KO} mutants. No obvious differences are apparent. Values are presented as average ± standard error of the mean (SEM). See Materials and Methods for quantification details. ns, $P > 0.05$ (Mann–Whitney–Wilcoxon test).

Data information: In all image panels, arrowhead points at cells positive for the relevant marker inside a clone. For full genotypes, see the Appendix.

 © 2017 The Authors

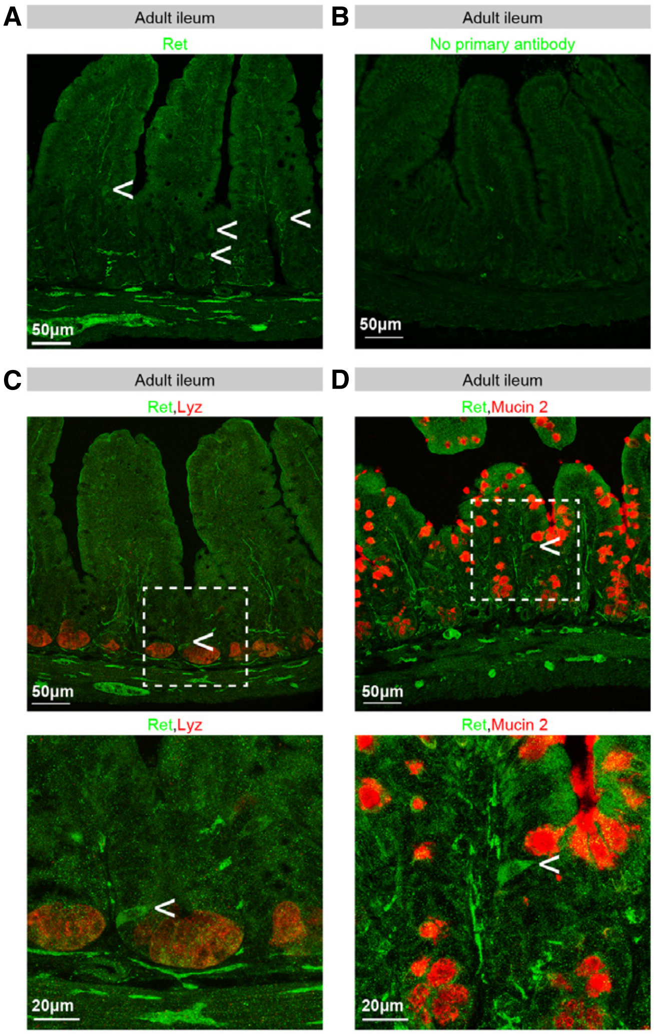

Figure EV2. Validation of Ret antibody and co-stainings with epithelial cell type-specific markers.

A Adult small intestinal tissue (ileum) stained with an anti-Ret antibody. Both neuronal fibres (at the bottom of the image) and scattered epithelial cells (arrows) are labelled.

B Neuronal and epithelial signals are absent from adult small intestinal tissue processed in parallel and subject to the same protocol as in for (A) except for incubation with the primary antibody. Only background, non-epithelial staining remains.

C Adult ileum section labelled with antibodies against Ret (green) and the Paneth cell marker lysozyme (Lyz, in red). Ret-positive cells (arrow) are not Lyz-positive.

D Adult ileum section labelled with antibodies against Ret (green) and the goblet cell marker mucin 2 (Muc2, red). Ret-positive cells (arrow) are not mucin 2-positive.

Data information: In (C and D), bottom panels are higher magnification images of the image regions boxed in the top panels.

© 2017 The Authors

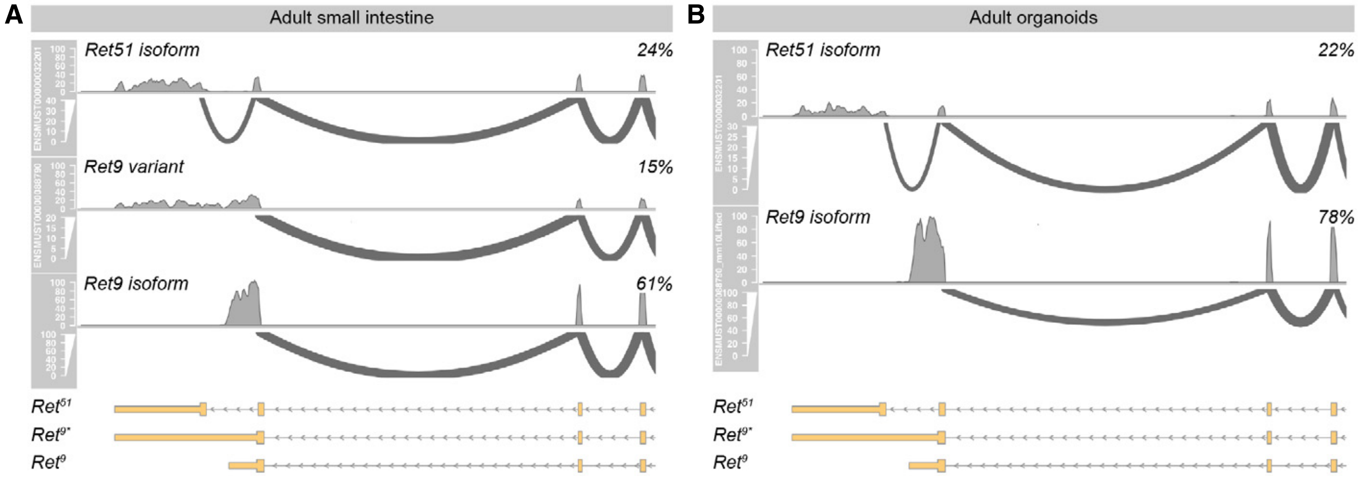

Figure EV3. Ret isoform usage in the intestine.

A In RNAseq samples obtained from adult small intestine, 24% of reads are assigned to the Ret51 isoform (Ensemble ID ENSMUST00000032201). The rest of reads (76%) map to two different transcript annotations which give rise to the same Ret9 isoform but differ slightly in their 3′UTR. Ret9 corresponds to the recently updated mm10 gene model for ENSMUST00000088790 (ENSMUST00000088790_mm10Update) whereas Ret9 variant corresponds to the previous mm9 gene model for ENSMUST00000088790.

B In RNAseq data obtained from epithelial organoids derived from adult small intestine, a similar isoform ratio is observed, but only one of the two Ret9 transcripts (Ret9, not Ret9 variant) is observed. See Materials and Methods for further details.

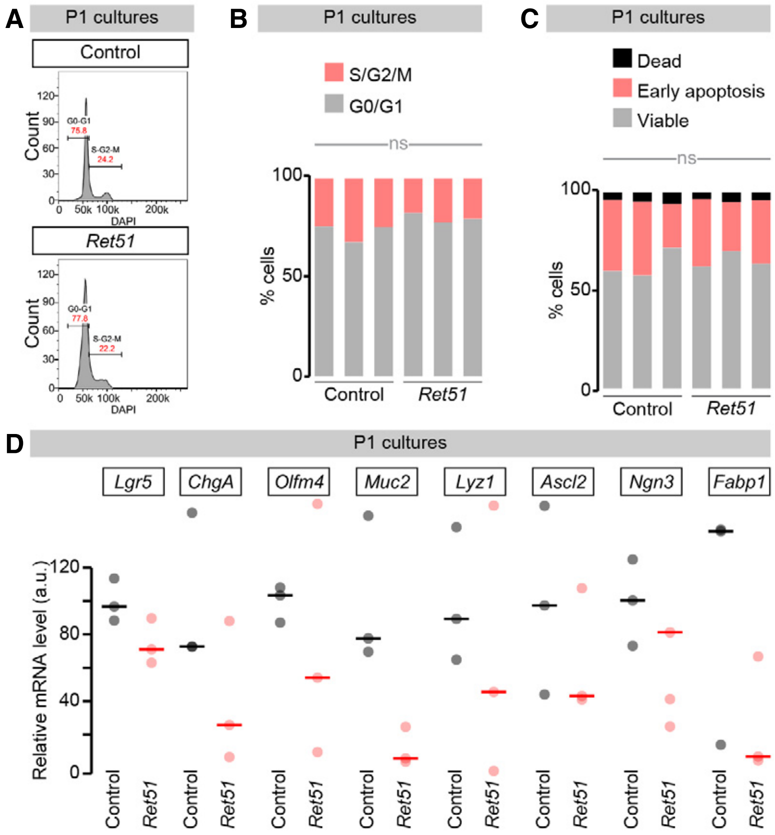

Figure EV4. Assessment of cell cycle state and viability of control and *Ret51* cells derived from epithelial cultures.

A Representative cell cycle histograms of cells dissociated from control and *Ret51* FEnS/organoid cultures.

B Quantifications of the percentage of cells in G0/G1 relative to S/G2/M in both genotypes. No obvious differences are apparent.

C Quantifications of the percentage of dead cells or cells undergoing apoptosis in both genotypes. No obvious differences are apparent. See Materials and Methods for quantification details.

D Transcript levels of epithelial cell differentiation markers relative to *Gapdh* transcript levels in RNA prepared from epithelial cultures derived from neonatal small intestinal tissue of *Ret51* mice or their control littermates, quantified in parallel to the *Axin2* transcript in Fig 6H.

Data information: Values are presented as averages, and each dot corresponds to an independent biological replicate.

 © 2017 The Authors
